# (*E*)-Methyl *N*′-[1-(4-methyl­phen­yl)ethyl­idene]hydrazinecarboxyl­ate

**DOI:** 10.1107/S160053680803184X

**Published:** 2008-10-04

**Authors:** Lu-Ping Lv, Wen-Bo Yu, Feng Wang, Wei-Wei Li, Xian-Chao Hu

**Affiliations:** a Department of Chemical Engineering, Hangzhou Vocational and Technical College, Hangzhou 310018, People’s Republic of China; b Research Center of Analysis and Measurement, Zhejiang University of Technology, Hangzhou 310014, People’s Republic of China

## Abstract

The title mol­ecule, C_11_H_14_N_2_O_2_, adopts a *trans* configuration with respect to the C=N bond. The dihedral angle between the benzene ring and the hydrazinecarboxyl­ate plane is 7.61 (16)°. In the crystal structure, mol­ecules are linked into centrosymmetric dimers by N—H⋯O hydrogen bonds and the dimers are linked together by C—H⋯π inter­actions.

## Related literature

For general background, see: Parashar *et al.* (1988 ▶); Hadjoudis *et al.* (1987 ▶); Borg *et al.* (1999 ▶). For a related structure, see Lv *et al.* (2008 ▶).
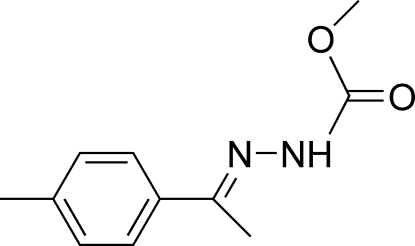


## Experimental

### 

#### Crystal data

C_11_H_14_N_2_O_2_
*M* *_r_* = 206.24
Monoclinic, 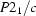

*a* = 11.5197 (3) Å
*b* = 5.5734 (6) Å
*c* = 17.3281 (2) Å
β = 94.193 (14)°
*V* = 1109.55 (12) Å^3^
*Z* = 4
Mo *K*α radiation
μ = 0.09 mm^−1^
*T* = 273 (2) K
0.24 × 0.22 × 0.20 mm

#### Data collection

Bruker SMART CCD area-detector diffractometer
Absorption correction: multi-scan (*SADABS*; Bruker, 2002 ▶) *T*_min_ = 0.978, *T*_max_ = 0.980
5502 measured reflections
1951 independent reflections
1209 reflections with *I* > 2σ(*I*)
*R*_int_ = 0.032

#### Refinement

*R*[*F* ^2^ > 2σ(*F* ^2^)] = 0.057
*wR*(*F* ^2^) = 0.172
*S* = 1.03
1951 reflections
138 parameters
3 restraints
H-atom parameters constrained
Δρ_max_ = 0.28 e Å^−3^
Δρ_min_ = −0.26 e Å^−3^

### 

Data collection: *SMART* (Bruker, 2002 ▶); cell refinement: *SAINT* (Bruker, 2002 ▶); data reduction: *SAINT*; program(s) used to solve structure: *SHELXS97* (Sheldrick, 2008 ▶); program(s) used to refine structure: *SHELXL97* (Sheldrick, 2008 ▶); molecular graphics: *SHELXTL* (Sheldrick, 2008 ▶); software used to prepare material for publication: *SHELXTL*.

## Supplementary Material

Crystal structure: contains datablocks I, global. DOI: 10.1107/S160053680803184X/cv2459sup1.cif

Structure factors: contains datablocks I. DOI: 10.1107/S160053680803184X/cv2459Isup2.hkl

Additional supplementary materials:  crystallographic information; 3D view; checkCIF report

## Figures and Tables

**Table 1:** Hydrogen-bond geometry (Å, °)

*D*—H⋯*A*	*D*—H	H⋯*A*	*D*⋯*A*	*D*—H⋯*A*
N2—H2*A*⋯O1^i^	0.86	2.12	2.944 (3)	162
C2—H2⋯*Cg*1^ii^	0.93	2.83	3.538 (3)	134

Symmetry codes: (i) 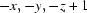; (ii) 
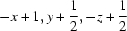
. *Cg*1 is the centroid of the C2–C7 benzene ring.

**Fig. 1.**
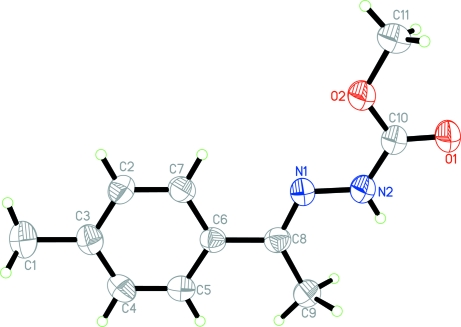
The molecular structure of the title compound, with the atom-numbering scheme. Displacement ellipsoids are drawn at the 40% probability level.

**Fig. 2.**
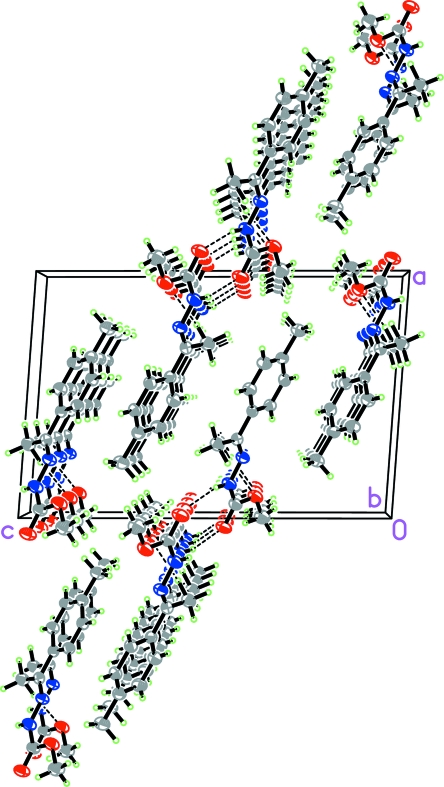
A packing diagram of the title compound. Hydrogen bonds are shown as dashed lines.

## supplementary crystallographic information

### Comment

Benzaldehydehydrazone derivatives have received considerable attention for a long time, due to their pharmacological activities (Parashar *et al.*, 1988) and their photochromic properties (Hadjoudis *et al.*,  1987). They are important intermidiates for 1,3,4-oxadiazoles, which have been reported to be versatile compounds with many useful properties (Borg *et al.*,  1999). As a further investigation of this type of derivatives, we report herein the crystal structure of the title compound.

The title molecule (Fig. 1) adopts a trans configuration with respect to the C═N double bond. The bond lengths and angles are comparable to those observed for (E)-methyl N'-[1-(4-methoxyphenyl)ethylidene]hydrazinecarboxylate (Lv *et al.*,  2008). All atoms of O1/O2/N1/N2/C8-C11 are coplanar within ±-0.093 (2)Å. The dihedral angle between the benzene (C2-C7) and O1/O2/N1/N2/C8-C11 planes is 7.61 (16)°.

In the crystal structure, intermolecular N—H···O hydrogen bonds (Table 1) link the molecules into centrosymmetric dimers (Fig. 2). A C—H···π contact (Table 1) between the benzene ring (centroid Cg1) and H atom of aromatic C2 further stabilizes the structure.

### Experimental

4-Methyl-acetophenone (1.34 g, 0.01 mol) and methyl hydrazinecarboxylate (0.90 g, 0.01 mol) were dissolved in stirred methanol (25 ml) and left for 5.5 h at room temperature. The resulting solid was filtered off and recrystallized from ethanol to give the title compound (yield 93%, m.p. 453-455 K). Single crystals of the title compound suitable for X-ray analysis were obtained by slow evaporation of an ethanol solution.

### Refinement

H atoms were positioned geometrically, with N-H = 0.86 Å and C-H = 0.93 and 0.96 Å for aromatic and methyl H, respectively, and constrained to ride on their parent atoms with U_iso_(H) = xU_eq_(C,N), where x = 1.5 for methyl H and x = 1.2 for all other H atoms. A rotating group model was used for the methyl groups.

### Crystal data

**Table:**

C_11_H_14_N_2_O_2_	*F*(000) = 440
*M**_r_* = 206.24	*D*_x_ = 1.235 Mg m^−^^3^
Monoclinic, *P*2_1_/*c*	Mo *K*α radiation, λ = 0.71073 Å
Hall symbol: -P 2ybc	Cell parameters from 1951 reflections
*a* = 11.5197 (3) Å	θ = 1.8–25.0°
*b* = 5.5734 (6) Å	µ = 0.09 mm^−^^1^
*c* = 17.3281 (2) Å	*T* = 273 K
β = 94.193 (14)°	Block, colourless
*V* = 1109.55 (12) Å^3^	0.24 × 0.22 × 0.20 mm
*Z* = 4	

### Data collection

**Table:**

Bruker SMART CCD area-detector diffractometer	1951 independent reflections
Radiation source: fine-focus sealed tube	1209 reflections with *I* > 2σ(*I*)
graphite	*R*_int_ = 0.032
φ and ω scans	θ_max_ = 25.0°, θ_min_ = 1.8°
Absorption correction: multi-scan (SADABS; Bruker, 2002)	*h* = −13→13
*T*_min_ = 0.978, *T*_max_ = 0.980	*k* = −6→6
5502 measured reflections	*l* = −19→20

### Refinement

**Table:**

Refinement on *F*^2^	Primary atom site location: structure-invariant direct methods
Least-squares matrix: full	Secondary atom site location: difference Fourier map
*R*[*F*^2^ > 2σ(*F*^2^)] = 0.057	Hydrogen site location: inferred from neighbouring sites
*wR*(*F*^2^) = 0.172	H-atom parameters constrained
*S* = 1.03	*w* = 1/[σ^2^(*F*_o_^2^) + (0.07*P*)^2^ + 0.5722*P*] where *P* = (*F*_o_^2^ + 2*F*_c_^2^)/3
1951 reflections	(Δ/σ)_max_ < 0.001
138 parameters	Δρ_max_ = 0.28 e Å^−^^3^
3 restraints	Δρ_min_ = −0.26 e Å^−^^3^

### Special details

**Table:**

Geometry. All esds (except the esd in the dihedral angle between two l.s. planes) are estimated using the full covariance matrix. The cell esds are taken into account individually in the estimation of esds in distances, angles and torsion angles; correlations between esds in cell parameters are only used when they are defined by crystal symmetry. An approximate (isotropic) treatment of cell esds is used for estimating esds involving l.s. planes.
Refinement. Refinement of F^2^ against ALL reflections. The weighted R-factor wR and goodness of fit S are based on F^2^, conventional R-factors R are based on F, with F set to zero for negative F^2^. The threshold expression of F^2^ > 2sigma(F^2^) is used only for calculating R-factors(gt) etc. and is not relevant to the choice of reflections for refinement. R-factors based on F^2^ are statistically about twice as large as those based on F, and R- factors based on ALL data will be even larger.

### Fractional atomic coordinates and isotropic or equivalent isotropic displacement parameters (Å^2^)

**Table:**

	*x*	*y*	*z*	*U*_iso_*/*U*_eq_	
C6	0.4332 (2)	−0.0332 (5)	0.39288 (14)	0.0447 (6)	
C5	0.5218 (2)	−0.2016 (5)	0.40434 (17)	0.0571 (8)	
H5	0.5107	−0.3340	0.4357	0.069*	
C8	0.3200 (2)	−0.0595 (5)	0.42808 (15)	0.0472 (7)	
C7	0.4558 (2)	0.1643 (5)	0.34621 (16)	0.0520 (7)	
H7	0.3994	0.2827	0.3379	0.062*	
C3	0.6471 (2)	0.0163 (5)	0.32361 (16)	0.0548 (7)	
C2	0.5594 (2)	0.1865 (5)	0.31268 (16)	0.0548 (7)	
H2	0.5712	0.3193	0.2817	0.066*	
C10	0.0517 (2)	0.2495 (5)	0.42066 (17)	0.0562 (7)	
C4	0.6258 (2)	−0.1771 (5)	0.37036 (18)	0.0632 (8)	
H4	0.6830	−0.2937	0.3792	0.076*	
C1	0.7601 (3)	0.0414 (7)	0.28540 (19)	0.0764 (10)	
H1A	0.7559	0.1786	0.2518	0.115*	
H1B	0.7732	−0.1002	0.2557	0.115*	
H1C	0.8230	0.0618	0.3243	0.115*	
C9	0.3016 (2)	−0.2711 (5)	0.47910 (17)	0.0602 (8)	
H9A	0.2698	−0.2178	0.5258	0.063*	
H9C	0.3746	−0.3499	0.4916	0.063*	
H9B	0.2484	−0.3812	0.4526	0.063*	
C11	−0.0088 (3)	0.5827 (6)	0.3454 (2)	0.0789 (10)	
H11A	−0.0725	0.5048	0.3167	0.118*	
H11B	0.0235	0.7033	0.3137	0.118*	
H11C	−0.0362	0.6564	0.3907	0.118*	
N2	0.13765 (19)	0.0920 (4)	0.44132 (14)	0.0613 (7)	
H2A	0.1257	−0.0182	0.4746	0.074*	
N1	0.24476 (18)	0.1054 (4)	0.40979 (13)	0.0548 (6)	
O2	0.07955 (17)	0.4080 (4)	0.36804 (12)	0.0690 (7)	
O1	−0.04293 (16)	0.2424 (4)	0.44769 (13)	0.0733 (7)	

### Atomic displacement parameters (Å^2^)

**Table:**

	*U*^11^	*U*^22^	*U*^33^	*U*^12^	*U*^13^	*U*^23^
C6	0.0480 (15)	0.0439 (15)	0.0421 (14)	0.0020 (12)	0.0018 (11)	−0.0030 (12)
C5	0.0586 (17)	0.0476 (17)	0.0660 (19)	0.0056 (13)	0.0102 (14)	0.0060 (14)
C8	0.0514 (15)	0.0426 (15)	0.0478 (15)	0.0001 (13)	0.0055 (12)	0.0006 (12)
C7	0.0509 (16)	0.0495 (17)	0.0559 (17)	0.0076 (13)	0.0054 (13)	0.0025 (14)
C3	0.0485 (16)	0.0588 (18)	0.0572 (18)	−0.0005 (14)	0.0052 (13)	−0.0077 (15)
C2	0.0564 (17)	0.0573 (18)	0.0515 (17)	−0.0049 (14)	0.0089 (13)	0.0058 (14)
C10	0.0485 (16)	0.0579 (18)	0.0631 (19)	−0.0015 (14)	0.0098 (14)	0.0062 (15)
C4	0.0537 (17)	0.0569 (19)	0.080 (2)	0.0145 (14)	0.0090 (15)	0.0013 (16)
C1	0.0543 (18)	0.095 (3)	0.082 (2)	−0.0016 (18)	0.0152 (16)	−0.004 (2)
C9	0.0566 (17)	0.0590 (19)	0.0662 (19)	0.0039 (14)	0.0116 (14)	0.0098 (15)
C11	0.071 (2)	0.070 (2)	0.096 (3)	0.0151 (18)	0.0100 (18)	0.0236 (19)
N2	0.0532 (14)	0.0602 (16)	0.0729 (16)	0.0043 (12)	0.0202 (12)	0.0171 (13)
N1	0.0459 (13)	0.0557 (15)	0.0641 (15)	0.0030 (11)	0.0133 (11)	0.0075 (12)
O2	0.0572 (12)	0.0718 (15)	0.0801 (15)	0.0104 (11)	0.0194 (11)	0.0241 (12)
O1	0.0492 (12)	0.0806 (16)	0.0922 (16)	0.0046 (11)	0.0205 (11)	0.0214 (13)

### Geometric parameters (Å, °)

**Table:**

C6—C5	1.390 (4)	C10—N2	1.351 (4)
C6—C7	1.402 (4)	C4—H4	0.9300
C6—C8	1.487 (3)	C1—H1A	0.9600
C5—C4	1.380 (4)	C1—H1B	0.9600
C5—H5	0.9300	C1—H1C	0.9600
C8—N1	1.286 (3)	C9—H9A	0.9600
C8—C9	1.498 (4)	C9—H9C	0.9600
C7—C2	1.370 (4)	C9—H9B	0.9600
C7—H7	0.9300	C11—O2	1.442 (3)
C3—C4	1.381 (4)	C11—H11A	0.9600
C3—C2	1.389 (4)	C11—H11B	0.9600
C3—C1	1.509 (4)	C11—H11C	0.9600
C2—H2	0.9300	N2—N1	1.388 (3)
C10—O1	1.218 (3)	N2—H2A	0.8600
C10—O2	1.326 (3)		
			
C5—C6—C7	116.5 (2)	C3—C1—H1A	109.5
C5—C6—C8	122.1 (2)	C3—C1—H1B	109.5
C7—C6—C8	121.3 (2)	H1A—C1—H1B	109.5
C4—C5—C6	121.6 (3)	C3—C1—H1C	109.5
C4—C5—H5	119.2	H1A—C1—H1C	109.5
C6—C5—H5	119.2	H1B—C1—H1C	109.5
N1—C8—C6	115.1 (2)	C8—C9—H9A	109.5
N1—C8—C9	125.8 (2)	C8—C9—H9C	109.5
C6—C8—C9	119.1 (2)	H9A—C9—H9C	109.5
C2—C7—C6	121.3 (3)	C8—C9—H9B	109.5
C2—C7—H7	119.3	H9A—C9—H9B	109.5
C6—C7—H7	119.3	H9C—C9—H9B	109.5
C4—C3—C2	117.0 (3)	O2—C11—H11A	109.5
C4—C3—C1	121.7 (3)	O2—C11—H11B	109.5
C2—C3—C1	121.3 (3)	H11A—C11—H11B	109.5
C7—C2—C3	121.9 (3)	O2—C11—H11C	109.5
C7—C2—H2	119.1	H11A—C11—H11C	109.5
C3—C2—H2	119.1	H11B—C11—H11C	109.5
O1—C10—O2	123.7 (3)	C10—N2—N1	121.1 (2)
O1—C10—N2	122.4 (3)	C10—N2—H2A	119.5
O2—C10—N2	113.9 (2)	N1—N2—H2A	119.5
C5—C4—C3	121.6 (3)	C8—N1—N2	117.8 (2)
C5—C4—H4	119.2	C10—O2—C11	115.8 (2)
C3—C4—H4	119.2		

### Hydrogen-bond geometry (Å, °)

**Table:**

*D*—H···*A*	*D*—H	H···*A*	*D*···*A*	*D*—H···*A*
N2—H2A···O1^i^	0.86	2.12	2.944 (3)	162
C2—H2···Cg1^ii^	0.93	2.83	3.538 (3)	134

Symmetry codes: (i) −*x*, −*y*, −*z*+1; (ii) −*x*+1, *y*+1/2, −*z*+1/2.
